# Supplementary material for: Hospitalisation trends in India from serial cross-sectional nationwide surveys: 1995 to 2014
Source: BMJ Open. 2017 Dec 19;7(12):e014188. doi: 10.1136/bmjopen-2016-014188 (PMC5770834; doi:10.1136/bmjopen-2016-014188)
Supplement: Supplementary data [file bmjopen-2016-014188supp001.pdf]

Table S1 Percent distribution of missing and deceased samples in NSS 1995–1996, NSS 2004 and NSS 2014, India

	NSS 1995–1996		NSS 2004		NSS 2014	
Background characteristics	N	%	N	%	N	%
			All ages			
Age	55	0.00	38	0.01	0	0.00
Sex	2	0.00	0	0.00	0	0.00
Place of residence	0	0.00	0	0.00	0	0.00
States	0	0.00	0	0.00	0	0.00
Economic status	0	0.00	0	0.00	0	0.00
N (including deceased persons)	633 405		385 055		335 499	
			60 years or more			
Marital status	27	0.12	0	0.00	0	0.00
Caste	21	0.05	9	0.01	0	0.00
Education	41	0.12	16	0.04	0	0.00
Economic dependency	780	2.29	602	1.45	11	0.01
Living arrangement	334	0.85	727	1.72	0	0.00
Physical mobility status	658	1.93	786	1.93	11	0.01
Self-rated health (SRH)	510	1.52	1,650	3.95	11	0.01
SRH compared to previous year	542	1.58	1,650	3.94	11	0.01
N (excluding deceased persons)	33 990		34 831		27 245	
% of hospitalised persons who died in 365 days reference period	1284	3.05	736	2.32	1152	2.18
N (including deceased persons)	35 274		35 567		28 397	

NSS, National Sample Survey; Caste in India is a social stratification of communities into 4 groups, namely scheduled castes (SCs), scheduled tribes (STs), other backward castes, and other castes. SC/STs are officially designated disadvantaged groups in India.
