# Supplementary material for: Hospitalisation trends in India from serial cross-sectional nationwide surveys: 1995 to 2014
Source: BMJ Open. 2017 Dec 19;7(12):e014188. doi: 10.1136/bmjopen-2016-014188 (PMC5770834; doi:10.1136/bmjopen-2016-014188)
Supplement: Supplementary data [file bmjopen-2016-014188supp002.pdf]

Table S2 List of diseases grouped according to Global Burden of Disease (GBD) study categorisation of diseases, 2013

Communicable diseases and nutritional disorders (CDs)	Non-communicable diseases and injuries (NCDs)
Tuberculosis	Neoplasms
STDs including HIV/AIDs	o Cancer and other tumours
Diarrhoeal diseases	Cardiovascular and circulatory diseases
o Cholera	o Heart disease, Hypertension
o Diarrhoea/dysentery/gastro-enteritis	o Rheumatic fever
o Amoebiosis	Chronic respiratory diseases
Respiratory infections and other common infectious disease	o Bronchial Asthma and related conditions
o Dengue/Influenza	Digestive diseases
o Pneumonia	o Gastrointestinal bleeding/piles
o Respiratory (including ear/nose/throat) ailments	o Gastritis/gastric/peptic ulcer
o Cough and acute bronchitis	o Cirrhosis/hydrocele
o Pleurisy	o Food poisoning
o Meningitis and viral encephalitis	Neurological disorder:
o Diptheria	o Cerebral stroke
o Pertussis/whooping cough	o Other diseases of nerves
o Tetanus	o Epilepsy/headache
o Measles/chicken pox/mumps/eruptive	o Nervous and general debility
Neglected tropical diseases and malaria	o Cerebral haemorrhage, thrombosis
o Filariasis	Mental and behavioural disorders
o Trachoma	Diabetes, urogenital, blood and endocrine diseases
o Worm infestation/Guinea worm	o Diabetes
o Leprosy	o Disease of kidney/urinary system/prostrate disorders
Neonatal and maternal disorders	o Gynaecological disorders
Nutritional deficiencies:	o Goiter/Thyroid disorders
o Anemia/bleeding disorders	Musculoskeletal disorders
o Under-nutrition	o Disorders of joints and bones
o Scurvy	o Locomotor disability
o Other malnutrition diseases (Beri-Beri , Ricket)	Other non-communicable diseases
Other communicable diseases and nutrition disorders:	Skin and subcutaneous diseases
o Hepatitis/Jaundice/diseases of liver	Sense organ diseases
o Fever of unknown origin/fever of short duration/malaria/typhoid	o Glucoma
	o Cataracts
	o Hearing loss, adult onset
	o Vision disorders, age related
	o Diseases of ear/nose/throat
	o Speech disability
	Oral disorders
	Accidents/injury/burns/fractures/poisoning
	Congenital anomalies
