# Supplementary material for: Hospitalisation trends in India from serial cross-sectional nationwide surveys: 1995 to 2014
Source: BMJ Open. 2017 Dec 19;7(12):e014188. doi: 10.1136/bmjopen-2016-014188 (PMC5770834; doi:10.1136/bmjopen-2016-014188)
Supplement: Supplementary data [file bmjopen-2016-014188supp003.pdf]

Table S3 Hospitalisation rates per 1000 (95% CI) for the older population by disease groups in the major states in NSS 1995–1996, NSS 2004 and NSS 2014, India

States	Hospitalisation rates per 1000 (95% CI)								
	NSS 1995–1996			NSS 2004			NSS 2014		
	All diseases	NCDs	CDs	All diseases	NCDs	CDs	All diseases	NCDs	CDs
Less developed	25.1 (22.3 to 27.9)	13.6 (12.1 to 15.1)	5.8 (4.0 to 7.6)	41.6 (38.4 to 44.9)	28.6 (25.8 to 31.4)	7.3 (6.2 to 8.4)	78.4 (71.3 to 85.5)	61.2 (54.6 to 67.8)	15.0 (12.7 to 17.2)
Assam	28.9 (20.4 to 37.3)	16.3 (10.1 to 22.4)	6.2 (2.2 to 10.2)	35.7 (24.0 to 47.5)	26.6 (15.4 to 37.7)	5.3 (3.0 to 7.7)	37.0 (24.0 to 50.0)	29.3 (16.6 to 42.0)	5.9 (3.3 to 8.5)
Bihar	15.4 (10.7 to 20.1)	8.1 (5.2 to 11.0)	4.4 (1.0 to 7.9)	28.1 (24.1 to 32.2)	19.4 (16.2 to 22.7)	4.7 (3.1 to 6.4)	52.6 (37.2 to 68.1)	44.9 (29.9 to 59.9)	6.5 (2.9 to 10.1)
Madhya Pradesh	29.7 (24.4 to 35.0)	16.7 (12.8 to 20.5)	7.4 (4.6 to 10.2)	47.2 (39.2 to 55.3)	34.7 (27.3 to 42.2)	9.4 (6.6 to 12.3)	101.2 (72.9 to 129.5)	80.0 (53.0 to 106.9)	18.9 (10.4 to 27.4)
Odisha	44.1 (21.2 to 66.9)	12.0 (7.9 to 16.1)	14.8 (-1.0 to 30.5)	42.0 (32.2 to 51.9)	21.0 (15.7 to 26.4)	14.6 (6.8 to 22.4)	79.6 (63.3 to 95.8)	57.7 (42.7 to 72.8)	20.2 (14.3 to 26.2)
Rajasthan	34.3 (25.6 to 43.1)	21.6 (14.5 to 28.8)	4.6 (2.5 to 6.7)	56.7 (45.9 to 67.5)	37.0 (30.0 to 44.0)	6.4 (3.5 to 9.3)	101.9 (88.6 to 115.2)	75.4 (64.0 to 86.8)	25.2 (18.5 to 31.9)
Uttar Pradesh	18.6 (15.1 to 22.0)	11.8 (9.5 to 14.2)	3.4 (1.2 to 5.6)	38.6 (32.0 to 45.2)	27.7 (21.6 to 33.8)	5.5 (4.1 to 6.9)	78.5 (65.5 to 91.4)	62.5 (50.8 to 74.2)	12.7 (8.6 to 16.7)
Jammu & Kashmir	34.3 (15.8 to 52.9)	19.4 (4.6 to 34.1)	8.7 (-1.8 to 19.3)	48.5 (36.4 to 60.6)	39.0 (28.0 to 50.0)	6.3 (1.9 to 10.7)	68.5 (50.4 to 86.7)	55.9 (39.8 to 71.9)	11.2 (2.9 to 19.6)

(…continues)

(…continued)

States	Hospitalisation rates per 1000 (95% CI)								
	NSS 1995–1996			NSS 2004			NSS 2014		
	All diseases	NCDs	CDs	All diseases	NCDs	CDs	All diseases	NCDs	CDs
More developed	70.9 (66.1 to 75.8)	41.7 (37.7 to 45.8)	12.7 (10.8 to 14.6)	104.6 (99.8 to 109.4)	74.6 (70.4 to 78.7)	17.1 (15.1 to 19.1)	134.3 (128.0 to 140.7)	109.7 (103.9 to 115.5)	21.1 (18.8 to 23.5)
Andhra Pradesh	47.0 (36.5 to 57.6)	30.8 (21.7 to 40.0)	6.2 (3.2 to 9.2)	65.9 (57.2 to 74.5)	54.4 (46.3 to 62.5)	5.8 (3.6 to 8.0)	111.2 (96.4 to 126.0)	94.1 (80.6 to 107.6)	12.9 (8.1 to 17.7)
Gujarat	45.9 (36.2 to 55.6)	18.4 (13.9 to 22.9)	19.3 (11.3 to 27.3)	102.5 (86.7 to 118.2)	64.6 (52.5 to 76.8)	27.3 (18.4 to 36.2)	123.7 (105.8 to 141.7)	98.0 (83.4 to 112.5)	24.9 (14.4 to 35.3)
Haryana	79.6 (57.0 to 102.1)	51.5 (33.4 to 69.6)	20.9 (9.1 to 32.7)	81.8 (57.2 to 106.5)	61.0 (38.5 to 83.5)	13.7 (5.4 to 22.0)	89.2 (71.5 to 106.8)	75.3 (58.7 to 91.9)	13.1 (7.1 to 19.1)
Karnataka	52.5 (37.8 to 67.2)	30.5 (18.4 to 42.6)	8.0 (2.6 to 13.3)	80.4 (68.2 to 92.6)	54.0 (44.7 to 63.3)	10.5 (5.7 to 15.3)	110.3 (96.9 to 123.7)	89.2 (76.9 to 101.4)	19.8 (14.6 to 25.1)
Kerala	200.5 (175.8 to 225.1)	110.5 (94.2 to 128.6)	39.0 (27.9 to 50.2)	279.1 (251.7 to 306.5)	190.5 (168.3 to 212.6)	47.0 (34.9 to 59.0)	281.3 (249.1 to 313.5)	216.2 (189.5 to 243.0)	51.5 (36.2 to 66.7)
Maharashtra	70.4 (60.3 to 80.5)	42.9 (35.0 to 50.9)	10.9 (7.6 to 14.2)	96.6 (85.0 to 108.2)	76.0 (65.1 to 86.8)	11.1 (8.0 to 14.1)	119.9 (103.1 to 136.7)	103.0 (86.5 to 119.4)	14.4 (11.1 to 17.7)
Punjab	45.6 (34.0 to 57.2)	21.7 (14.0 to 29.3)	4.7 (1.7 to 7.7)	80.7 (63.2 to 98.2)	58.8 (43.7 to 73.8)	12.5 (5.1 to 19.8)	103.7 (80.0 to 127.5)	89.5 (66.6 to 112.5)	12.7 (6.8 to 18.6)
Tamil Nadu	72.7 (52.7 to 92.7)	52.3 (32.8 to 71.8)	7.7 (5.2 to 10.2)	105.6 (92.0 to 119.2)	71.9 (60.9 to 82.9)	23.1 (15.8 to 30.4)	138.1 (118.5 to 157.7)	115.3 (96.6 to 134.0)	22.1 (16.3 to 27.8)
West Bengal	41.5 (33.0 to 50.1)	22.1 (17.4 to 26.9)	8.0 (2.3 to 13.7)	68.5 (59.5 to 77.4)	46.7 (38.8 to 54.6)	11.5 (8.4 to 14.6)	109.4 (98.1 to 120.7)	86.3 (76.0 to 96.6)	18.7 (14.3 to 23.1)
India	49.7 (46.8 to 52.6)	28.7 (26.5 to 31.0)	9.5 (8.2 to 10.8)	76.4 (73.4 to 79.4)	54.0 (51.4 to 56.5)	12.7 (11.5 to 13.9)	109.9 (105.2 to 114.5)	88.5 (84.2 to 92.8)	18.4 (16.8 to 20.1)

NSS, National Sample Survey; CI, Confidence intervals.
