# Supplementary material for: Hospitalisation trends in India from serial cross-sectional nationwide surveys: 1995 to 2014
Source: BMJ Open. 2017 Dec 19;7(12):e014188. doi: 10.1136/bmjopen-2016-014188 (PMC5770834; doi:10.1136/bmjopen-2016-014188)
Supplement: Supplementary data [file bmjopen-2016-014188supp004.pdf]

Table S4 Hospitalisation rates per 1000 (95% CI) in public hospitals among the older population in the major states in NSS 1995–1996, NSS 2004 and NSS 2014, India

	Hospitalisation rates per 1000 (95% CI) in public hospitals								
	NSS 1995–1996			NSS 2004			NSS 2014		
States	Non-poor	Poor	Total	Non-poor	Poor	Total	Non-poor	Poor	Total
Less developed	53.3 (45.6 to 60.8)	64.8 (56.0 to 72.7)	57.1 (51.3 to 62.6)	38.7 (33.6 to 44.2)	59.5 (54.9 to 63.9)	48.9 (45.0 to 52.9)	36.0 (30.4 to 41.9)	55.0 (48.9 to 60.9)	45.2 (40.9 to 49.6)
Assam	78.8 (61.2 to 89.8)	67.2 (33.3 to 89.4)	76.0 (60.1 to 86.9)	47.7 (25.4 to 70.9)	83.8 (66.7 to 93.0)	64.4 (44.9 to 80.1)	78.3 (65.3 to 87.4)	86.6 (72.0 to 94.2)	82.3 (72.3 to 89.2)
Bihar	35.5 (19.6 to 55.4)	22.9 (9.1 to 46.7)	31.3 (18.4 to 48.0)	14.3 (9.5 to 20.9)	27.5 (19.2 to 37.7)	21.3 (16.0 to 27.6)	20.5 (11.9 to 33.0)	42.8 (32.6 to 53.6)	28.8 (20.3 to 39.1)
Madhya Pradesh	43.6 (33.3 to 54.4)	72.0 (56.5 to 83.6)	51.4 (42.2 to 60.5)	35.1 (26.8 to 44.4)	67.0 (53.1 to 78.4)	51.6 (43.1 to 60.0)	24.5 (14.8 to 37.7)	48.1 (31.3 to 65.3)	37.2 (26.2 to 49.8)
Odisha	92.6 (81.6 to 97.3)	93.4 (84.5 to 97.3)	92.9 (85.5 to 96.6)	74.6 (61.2 to 84.6)	86.9 (76.3 to 93.2)	81.1 (72.6 to 87.5)	71.0 (58.8 to 80.8)	85.8 (76.9 to 91.6)	79.2 (72.5 to 84.7)
Rajasthan	60.7 (44.1 to 75.1)	44.7 (23.7 to 67.7)	55.6 (42.1 to 68.4)	52.7 (39.0 to 66.0)	70.9 (60.3 to 79.7)	59.9 (50.0 to 69.1)	48.8 (40.5 to 57.2)	66.5 (57.2 to 74.7)	58.9 (52.4 to 65.0)
Uttar Pradesh	30.9 (22.8 to 40.4)	54.2 (38.2 to 69.4)	38.6 (30.2 to 47.8)	24.7 (17.4 to 33.9)	44.7 (36.7 to 53.0)	34.3 (27.7 to 41.5)	26.8 (18.5 to 37.0)	30.8 (23.0 to 39.9)	28.4 (22.4 to 35.3)
Jammu & Kashmir	94.5 (82.7 to 98.4)	99.6 (97.1 to 100.0)	97.7 (93.6 to 99.2)	92.6 (84.6 to 96.6)	85.9 (71.3 to 93.8)	89.1 (80.7 to 94.0)	87.1 (73.9 to 94.1)	94.9 (86.7 to 98.1)	92.6 (86.2 to 96.1)

(….continues)

(….continued)

States	Hospitalisation rates per 1000 (95% CI) in public hospitals								
	NSS 1995–1996			NSS 2004			NSS 2014		
	Non-poor	Poor	Total	Non-poor	Poor	Total	Non-poor	Poor	Total
More developed	27.2 (23.6 to 31.1)	52.4 (46.9 to 57.8)	38.5 (35.0 to 42.1)	28.1 (25.0 to 31.3)	42.6 (39.4 to 45.8)	36.1 (33.9 to 38.4)	20.7 (18.0 to 23.6)	41.1 (38.2 to 44.1)	31.6 (29.5 to 33.8)
Andhra Pradesh	16.3 (10.0 to 25.5)	42.2 (27.9 to 57.9)	24.6 (17.6 to 33.2)	24.1 (15.9 to 34.7)	38.8 (30.8 to 47.4)	32.0 (26.2 to 38.5)	14.6 (8.7 to 23.3)	29.9 (22.8 to 38.0)	22.6 (17.7 to 28.3)
Gujarat	27.2 (15.9 to 42.5)	64.9 (47.1 to 79.3)	40.6 (30.0 to 52.2)	17.7 (11.2 to 26.8)	33.6 (24.4 to 44.3)	25.4 (19.5 to 32.3)	16.7 (10.3 to 26.0)	33.6 (26.0 to 42.0)	24.9 (19.5 to 31.2)
Haryana	39.8 (24.7 to 57.0)	25.2 (10.8 to 48.4)	33.3 (22.0 to 46.8)	20.8 (11.5 to 34.6)	18.2 (9.2 to 33.0)	19.6 (12.5 to 29.2)	6.9 (3.8 to 12.4)	52.9 (39.0 to 66.3)	29.7 (21.3 to 39.8)
Karnataka	33.0 (19.6 to 49.9)	46.3 (27.5 to 66.3)	35.1 (23.1 to 49.5)	20.8 (12.9 to 31.6)	51.4 (40.6 to 62.0)	35.4 (28.3 to 43.2)	26.5 (16.3 to 40.1)	28.5 (22.4 to 35.5)	27.8 (22.1 to 34.2)
Kerala	21.1 (14.4 to 29.9)	55.1 (47.2 to 62.8)	42.0 (35.9 to 48.4)	26.9 (20.2 to 34.9)	41.0 (35.0 to 47.3)	35.6 (31.0 to 40.5)	20.3 (14.4 to 27.8)	49.5 (42.3 to 56.7)	33.8 (28.8 to 39.3)
Maharashtra	15.2 (9.9-22.8)	35.8 (26.3 to 46.5)	25.1 (19.4 to 31.9)	22.7 (15.6 to 31.7)	36.2 (29.0 to 44.1)	30.7 (25.4 to 36.5)	9.3 (6.2 to 13.7)	29.7 (22.3 to 38.2)	20.5 (15.7 to 26.3)
Punjab	35.8 (22.9 to 51.1)	41.8 (22.7 to 63.7)	38.3 (27.0 to 51.0)	32.4 (20.0 to 47.9)	25.2 (14.4 to 40.2)	29.4 (20.4 to 40.3)	22.3 (7.5 to 50.6)	24.8 (16.1 to 36.2)	23.6 (13.8 to 37.3)
Tamil Nadu	21.5 (14.1 to 31.5)	69.4 (49.7 to 83.9)	43.2 (29.3 to 58.2)	16.7 (11.6 to 23.3)	43.5 (34.8-52.6)	33.6 (27.7 to 40.1)	13.6 (9.2 to 19.7)	40.7 (32.9 to 49.1)	30.8 (25.7 to 36.4)
West Bengal	62.3 (51.5-72.0)	83.0 (65.1 to 92.7)	69.0 (59.6 to 77.1)	60.2 (51.6 to 68.3)	82.1 (75.0 to 87.5)	69.0 (63.2 to 74.2)	49.8 (43.2 to 56.4)	72.1 (63.4 to 79.4)	61.0 (55.9 to 65.9)
India	34.1 (30.4 to 37.9)	54.6 (49.9 to 59.2)	42.7 (39.7 to 45.8)	30.9 (28.3 to 33.6)	46.3 (43.6 to 49.1)	39.2 (37.3 to 41.2)	25.8 (23.2 to 28.4)	45.2 (42.5 to 47.9)	35.9 (33.9 to 37.8)

NSS, National Sample Survey; CI, Confidence intervals.
